# Burden of non-communicable diseases among Syrian refugees: a scoping review

**DOI:** 10.1186/s12889-019-6977-9

**Published:** 2019-05-24

**Authors:** Farah Naja, Hibeh Shatila, Maria El Koussa, Lokman Meho, Lilian Ghandour, Shadi Saleh

**Affiliations:** 1 Nutrition and Food Sciences Department, Faculty of Agriculture and Food Sciences , American University of Beirut, Beirut, Lebanon; 2 Global Health Institute, American University of Beirut, Beirut, Lebanon; 3 University Libraries, American University of Beirut, Beirut, Lebanon; 4 Department of Epidemiology and Population Health, Faculty of Health Sciences, Beirut, Lebanon; 5 Department of Health Management and Policy, Faculty of Health Sciences, Beirut, Lebanon

**Keywords:** Syrian refugees, Non-communicable diseases, Conflict, Health

## Abstract

**Background:**

The scarcity of evidence-based research on non-communicable diseases (NCDs) among Syrian refugees has hampered efforts to address the high burden of these diseases in host countries. The objective of this study is to examine published research on NCDs among Syrian refugees in order to inform future research, practice, programs, and policy. .

**Methods:**

Using the scoping review framework proposed by Arksey et al., 17 different databases were searched to identify studies reporting on NCDs among Syrian refugees. The number of relevant documents found was 34, with the earliest going back to 2013—2 years after the beginning of the Syrian conflict.

**Results:**

The majority of these documents were descriptive in nature and only two studies addressed the effectiveness of interventions in the management of NCDs. No studies investigated the prevention of these diseases. Furthermore, only 7 studies addressed the host community and only one research article, conducted in Lebanon, included subjects from the host community. The increasing number of documents over the past 5 years illustrates a growing interest in studying NCDs among Syrian refugees. Examination of the papers showed high prevalence of NCDs among Syrian refugees as well as unmet healthcare needs.

**Conclusion:**

The findings of this review highlighted the dire need for further research on the burden of NCDs among Syrian refugees. Future studies should diversify research design to include interventions, address the host community in addition to the refugees, tackle prevention as well as treatment of NCDs, and explore strategies to enhance the resilience of the host country’s health system while ensuring quality of care for NCDs. The increasing momentum for research found in this review presents an opportunity to fill current knowledge gaps, which could result in preventing, controlling and ultimately reducing the burden of NCDs among Syrian refugees and their host communities.

**Electronic supplementary material:**

The online version of this article (10.1186/s12889-019-6977-9) contains supplementary material, which is available to authorized users.

## Background

Worldwide, approximately 70% of total deaths are attributed to non-communicable diseases (NCDs), mainly cardiovascular diseases (CVD), cancers, diabetes and chronic respiratory diseases [1]. This significant burden of NCDs is disproportionally distributed between high and low middle-income countries (LMICs), whereby over 75% of NCDs’ deaths occur in LMICs [2], which are currently undergoing a nutrition and disease transition as a result of increased globalization and modernization [3]. Of further concern is the fact that 29% of NCD deaths in LMICs occur among people under the age of 60, compared to 13% in high-income countries [4]. In addition to NCDs, infectious diseases are also prevalent in many LMICs hence creating a double burden of disease and posing a heavy burden on health systems and population health in these countries [5]. The burden of NCDs is further amplified in LMICs that are experiencing political instability and/or receiving a large number of refugees fleeing wars in neighboring countries [6]. In Syria, a LMIC which has been experiencing war and conflict since 2011, NCDs are estimated to account for 45% of all deaths, according to the latest WHO report [7].

Today, the Syrian refugee crisis is considered to be the largest displacement emergency in the world [8]. Up until 2017, more than 5.4 million people have been displaced as result of the Syrian war. Filippo Grandi (UN Higher Commissioner for Refugees) described the situation in Syria as ‘The biggest humanitarian and refugee crisis of our time’ [8]. Although refugees have fled to a few countries in Europe and North America, the majority of Syrian refugees sought refuge in neighboring countries, such as Lebanon, Jordan and Turkey [9, 10]. Lebanon hosts the largest refugee population per capita (997,905 registered individuals) while Jordan and Turkey host 655,624 and 3,424,237 refugees respectively [10]. Such a heavy influx of refugees has placed enormous pressures on the economies, societies, and infrastructure of these countries, most notably on the health systems. In order to cope with the urgent healthcare requirements, the health systems in these countries struggled to address the acute health needs of the refugees and have often neglected the care for NCDs’ management and prevention [11]. Refugees are particularly susceptible to NCDs, with a few studies documenting a shift from acute to chronic disease burden [12–14]. Although the causes of such a shift are not fully understood, it is postulated that the stress which results from leaving one’s home increases the susceptibility to many chronic diseases such as hypertension, diabetes and many types of cancer [13, 15]. Furthermore, refugees in many instances undergo lifestyle changes affecting dietary intake and activity levels which may increase the risk of NCDs [16, 17]. A recent study examining the prevalence of NCDs among non-camp Syrian refugees in northern Jordan showed that 21.8% of adults suffered from at least one NCD, with hypertension and Type 2 Diabetes being the most common (14 and 9.2%, respectively) [18].

In view of the unremitting nature of the crisis in Syria and the high prevalence of NCDs burden among both Syrian refugees and host communities, there is an imminent need for evidence based interventions and recommendations to address the burden of these diseases, both at the prevention and management levels. Although previous research has suggested a few successful examples of interventions in the context of NCDs in humanitarian crises [19–21], their findings have limited applicability to Syrian refugees given the distinct NCDs’ profiles in this population and the specificities of their humanitarian crisis.

A recent systematic review of the effectiveness of interventions for NCDs in humanitarian crises concluded that interventions that included standardization of care and improvement of patient follow up yielded positive results. However this systematic review also raised concerns that quantity and quality of evidence on the topic were extremely limited and that substantially more research is needed [22]. According to a seminal paper, new research should not be done unless, at the time it is initiated, the questions it proposes to address cannot be answered satisfactorily with existing evidence [23]. Therefore, the main objective of this review is to examine published research on NCDs among Syrian refugees in order to inform future research, practice, programs, and policy.

## Methods

This scoping review followed the Arksey et al. framework for knowledge synthesis of research which consists of five iterative steps: (1) identifying the research question;(2) identifying relevant studies; (3) selecting relevant studies; (4) data charting and finally; (5) collating, summarizing, and reporting the results [24]. Unlike systematic reviews, scoping reviews provide an overview of the state of the research activity rather than evaluating its quality. The methodology used in scoping reviews is rather flexible allowing for the inclusion of grey literature as well as studies with diverse study designs [25]. In this review, a Syrian refugee is defined as a Syrian citizen who, owing to ongoing war in Syria, was compelled to leave his place of habitual residence in order to seek refuge [26]. The host community is defined as “the country of asylum and the local, regional and national governmental, social and economic structures within which refugees live. Urban refugees live within host communities with or without legal status and recognition by the host community. In the context of refugee camps, the host community may encompass the camp, or may simply neighbor the camp but have interaction with, or otherwise be impacted by, the refugees residing in the camp” [27].

### Identification of the research question

The primary research question that guided this study was ‘what has been published with regards to NCDs among Syrian refugees? more specifically, ‘what is the current evidence with regards to available *interventions aiming to address* NCDs *in this population.* This research question will lead to the identification of gaps and opportunities in the existing knowledge of NCDs’ burden among Syrian refugees.

### Identifying relevant studies

Studies reporting on NCDs among Syrian refugees were identified by searching 17 different databases, including Academic Search Complete (by EBSCO), CINAHL Plus with Full Text, Directory of Open Access Journals (which includes over 250 periodicals published in the Arab world), Embase, Global Health Library, Historical Abstracts, ProQuest Central, PsycINFO, PubMed, ScienceDirect, Scopus, Sociological Abstracts, Web of Science, and Worldwide Political Science Abstracts, as well as three Middle Eastern studies related databases—AlManhal, E-Marefa, and Middle Eastern & Central Asian Studies. The search strategy consisted of two broad queries, in the title, abstract, and keyword fields, as follows:

1. (Syria* OR Syrie*) AND refuge*
2. (Syria* OR Syrie*) AND (asylum* OR camp OR camps OR deport* OR diaspora OR displaced OR emigrant* OR emigre* OR escapee* OR evacuee* OR exile* OR expatriate* OR immigrant* OR runaway* OR survivor* OR transient* OR victim*) AND (conflict* OR war OR wars)

The two searches, which were carried out in late September 2017, targeted all types of literature in Arabic, English and French, and the union of both resulted in a total of 3630 documents; the number was reduced to 1500 after removing all duplicate records.

### Selection of relevant articles

For a document to be included in the study, it had to meet the following criteria:

- The main topic of the document was Syrian refugees, regardless of their location
- The document had to be a journal article, conference paper, book chapter, short paper, report, or case study
- The study had to focus on any NCD, such as CVD (e.g., heart attacks and stroke), cancers, chronic respiratory diseases (e.g., chronic obstructive pulmonary disease (COPD)), and diabetes or any of the NCDs’ risk factors in relation to Syrian refugees
- The study had to include an outcome related to NCDs and/ or their complications
- The document had to be published from 2011 on, that is, from the time the Syrian conflict started (March 2011) [8]

A document was excluded from the study if it was an authored book, editorial, letter, note, abstract, news piece, or book review published before 2011 or was in a language other than Arabic, English, or French.

Two reviewers (HC and RA) independently reviewed the titles and abstracts and screened the documents for eligibility. The full text versions of eligible documents were retrieved and independently screened by the two reviewers to determine whether they met the inclusion criteria. Disagreements about whether the inclusion criteria were met were resolved through discussion with a third reviewer (FN).

### Data charting

A comprehensive data extraction form was developed using an inclusive approach in order to avoid omitting any findings of potential value to the scoping review. The development of this form was followed by an iterative review process by an expert panel consisting of a health system expert, an epidemiologist and a statistician. Following its development and review, the data extraction form was pilot tested on five randomly selected documents. The panel further reviewed this form in light of the pilot test results. The final version of the form contained descriptive information about: (1) general information; (2) authors’ affiliations; (3) country where the study took place; (4) NCDs and their risk factors; (5) study design; (6) study population; (7) funding source; and (8) health system indicators addressed. The data extraction form can be found in Additional file 1.

### Collating, summarizing, and reporting the results

Data entry and analysis were conducted using the Statistical Package for the Social Sciences (SPSS) software version 23.0 for Windows [28]. After entry, data files were checked for completeness (i.e. missing entries). Descriptive characteristics of the documents included in this study were presented as proportions, n (%).

## Results

### Characterization of documents addressing NCDs’ research among Syrian refugees

**Fig. 1:**
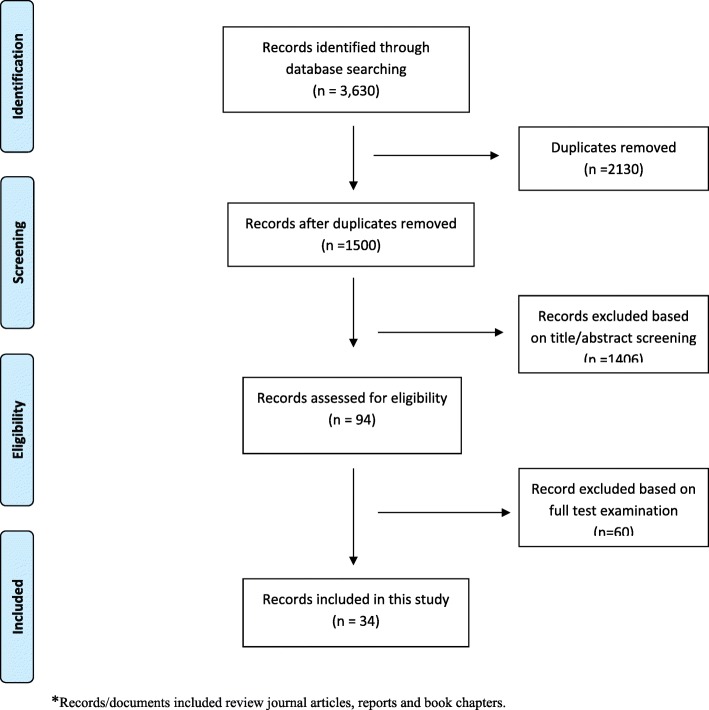
Flowchart describing the selection of records/documents to be included in this study

Of the 1500 documents screened for inclusion based on the title and abstract, only 94 were found relevant (i.e. met the eligibility criteria). The main reason for excluding the 1406 documents was that they did not address NCDs. The examination of the full text of the 94 documents resulted in 34 that were eligible for inclusion in the study (see Fig. 1 for more details).

**Fig. 2:**
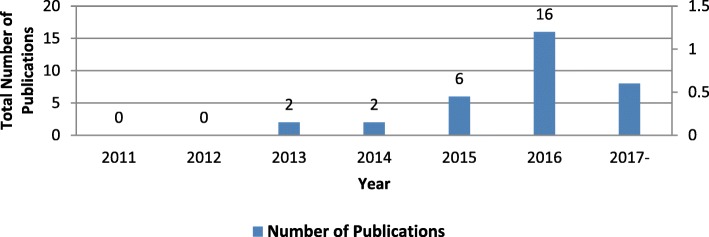
Time trend in the numbers of papers on NCDs’ research among Syrian refugees (2011–2016)

The characteristics of the 34 documents included in this study are presented in Table 1. The documents included in this study addressed NCDs among Syrian refugees mainly in countries neighboring Syria: Jordan (15), Lebanon (9), Turkey (3) and Iraq (2). The majority of the studies were either cross-sectional (17) or reviews (5), with only two intervention-based. Few studies (7) addressed the host community, in addition to Syrian refugees. Samples in 11 of the studies were recruited mainly from healthcare facilities or the local community (5). The studies included all age groups with greater focus on adolescents, adults and older adults as compared to pregnant and breastfeeding women, infants and children. While 25 of the studies tackled NCDs in general, diabetes was the most commonly addressed NCDs (21), followed by CVD and stroke (17), and chronic lung dysfunction/asthma/COPD (16). Cancer was the least studied NCD (7). Almost one in three studies addressed an infrastructural factor for NCDs, such as access to healthcare or cost. The majority of the studies was funded by INGOs and NGOs (10) and by the industry (5), while government-funded research was the least reported (1). Half of the studies addressed a health system issue, mainly health service delivery (15) or health financing (6). Health information systems and health workforce were examined in 3 and 1 of the studies, respectively. Figure 2 illustrates the number of studies per year, showing a steady increase since 2013 with the largest number of documents published in 2016 (16).

**Table 1:** Characterization of documents addressing NCDs’ research among Syrian refugees (2011–2017). (*n* = 34)

	n(%)^b^
Document type	
Journal article	24 (70.6)
Report	7 (20.6)
Book chapter	3 (8.8)
Authors’ affiliations^a^	
Academic institutions only	15 (44.1)
Academic and other institutions^b^	10 (29.4)
INGO/NGOs (only)	3 (8.8)
Hospital (only)	1 (2.9)
N/A (reports)	5 (14.7)
Countries of institutions leading in research on SR and NCD (according to corresponding author)	
USA	8 (23.5)
Jordan	6 (17.6)
UK	3 (8.8)
Turkey	2 (5.9)
Slovenia	2 (5.9)
Other (Belgium (1), Denmark(1), Greece(1), Iran(1), Israel(1), Slovakia(1), Spain(1), Switzerland(1))	8 (23.5)
N/A ^d^	5 (14.7)
Study location^d^	
Jordan	15 (38.5)
Lebanon	9 (23.1)
Turkey	3 (7.7)
Iraq	2 (5.1)
Greece	1 (2.6)
Belgium	1 (2.6)
Slovenian-Croatian border	1 (2.6)
Other ^d^	7 (17.9)
Study design	
Cross-sectional	17 (50.0)
Review	5 (14.7)
Interventions	2 (5.9)
NA (reports and book chapters)	10 (29.4)
Sample population studied ^c^	
Syrian refugees (no other refugees)	22 (64.7)
Other refugees (in addition to Syrians)	12 (35.3)
Sample include IDPs	3 (8.8)
Sample include host community	7 (20.6)
Living conditions of the study population^c^	
Decentralized accommodation	12 (30.8)
Formal tented settlement/initial reception centers/Collective accommodation center	4 (10.3)
Informal tented settlements	3 (7.7)
Not specified	19 (48.7)
On the Balkan route	1 (2.6)
Sample recruitment^c^	
Healthcare facility (Hospital, PHC)	11 (31.4)
Community (population-based)	5 (14.3)
NGO Facility	3 (8.6)
Refugee camps	1 (2.9)
Schools	0 (0.0)
N/A (did not include recruitment of study participants)	15 (42.9)
Methods for data collection^c^	
Chart/record extraction	10 (23.3)
Face-to-face household/site survey	13 (20.2)
Telephone interview	5 (11.6)
Online survey	0 (0.0)
N/A	15 (34.9)
Age of the study population^c^	
Pregnant and breastfeeding	11 (8.2)
Infant and child (< 2 years)	15 (11.2)
Children (2–9 years)	17 (12.7)
Children and Adolescents (> 10 and > 18)	18 (13.4)
Young adults (18–25)	20 (14.9)
Adults (18–50)	26 (19.4)
Older Adults (>50)	27 (20.2)
Sex of study population	
Both males and females	34 (100)
Males or females only	0 (0.0)
Types of NCDs^c^	
NCD in general	25 (29.1)
Diabetes	21 (24.4)
CVD and stroke	17 (19.8)
Chronic lung dysfunction/Asthma/COPD	16 (18.6)
Cancer	7 (8.1)
Risk factors addressed^c^	
Structural Factors/ Access to care/cost	25 (30.5)
Hypertension	22 (26.8)
Nutrition/ diet	9 (11.0)
High blood glucose	6 (7.3)
Social determinants	6 (7.3)
Obesity/Overweight/BMI	4 (4.9)
Tobacco	4 (4.9)
High blood cholesterol or hyperlipidemia	1 (1.2)
Other ^e^	5 (6.1)
Theme in relation to NCDs^c^	
Policy/ health system/ insurance/ pension plans	21 (41.2)
Prevalence/Incidence/distribution/descriptive/diagnosis	23 (45.1)
Etiology/risk factor/determinants/analytical/management and complications	5 (9.8)
Prevention and Control	2 (3.9)
Funding source ^c^	
INGO/NGO	10 (29.4)
Private/industry	5 (14.7)
Governmental	1 (2.9)
No funding	2 (5.9)
Not applicable	16 (47.1)
Health system indicators	
Articles that did not address any health system indicators	17 (50.0)
Articles addressing at least one indicator	17 (50.0)
Type of health system indicators addressed ^c^	
Health service delivery	15 (42.9)
Health financing	6 (17.1)
Health information system	3 (8.6)
Health workforce	1 (2.9)

^**a**^The affiliations of all contributing authors were considered

^b^Academic-NGOs 8(80.0), Academic-Government 1(10.0) Academic-Government -NGOs 1(10.0)

^c^Multiple answers were applicable

^d^Other include 2 articles on refugees in Europe, 1 article on refugees in Europe and Middle east, and 1 article on refugees all over the word and 3 no country specified

^e^Other includes family history, genetic predisposition, alcohol and other Substance abuse, physical inactivity, awareness

### Synthesis of main findings

Table 2 presents a summary of the characteristics and main findings of the 19 studies determined to be ‘original’ research (a more detailed description of these studies is included in Additional file 2). A thorough examination of the objectives and results of these 19 studies revealed two overarching themes: prevalence of NCDs and healthcare needs for NCDs’ patients. The extraction of the two themes in the manuscript followed a thematic analysis, more specifically an inductive type of thematic analysis [48].

**Table 2:** Summary of the characteristics and main findings of studies addressing NCDs among Syrian refugees (*n* = 19)^a, b^

Title	Brief description	Findings: Prevalence of NCDs among SR	Findings Healthcare needs of SR in relation to NCDs
The Provision of Health Services in Jordan to Syrian Refugees Al-Fahoum et al. [29]	Cross-sectional survey of adult SR (*n* = 120), camp setting in Jordan	HT: 41% in men and 30% among women	46% reported receiving ‘bad’ healthcare (54% men and 42% women) and 75% reporting insufficient healthcare
Prevalence and care-seeking for chronic diseases among Syrian refugees in Jordan Doocy et al. [30]	Cross-sectional survey of adults SR (*n* = 1550 HH), in non-camp setting in Jordan	One in two HH reported NCD (50.3%). HT (10.7%), arthritis (7.1%), DM (6.1%), CVD (4.1%) and COPD (2.9%)	Among cases who did not seek healthcare, barriers cited were: cost, not knowing where to go, could not afford transportation and no trust in the provider of care
Health service access and utilization among Syrian refugees in Jordan Doocy et al. [31]	Cross-sectional survey of adult SR (*n* = 1550 HH, 9580 subjects) outside camps in Jordan	N/A	86.1% of HH reported an adult sought medical care the last time it was needed. 51.5% of services were sought from public sector, 38.7% private, and 9.8% in charity/NGO facilities. 51.8% of HH reported out-of pocket expenditures for medical care.
Chronic Diseases, Lack of Medications, and Depression Among Syrian Refugees in Jordan, 2013–2014 Gammoush et al. [32]	Cross-sectional survey of adult SR (*n* = 765) (> 18 yrs) attending Caritas in 6 cities [self-reported], living in urban areas in Jordan	N/A	71.9% reported not having enough medications
Health Service Utilization among Syrian Refugees with Chronic Health Conditions in Jordan Doocy et al. [33]	Cross-sectional survey of adults SR (*n* = 1550 HH) in non-camp setting in Jordan	Prevalence of NCD (21.1%) [reasons for needing healthcare]	51.5% sought care from public sector, 38.7% private, 9.8 NGO/charity. Cost was a main barrier for not seeking care Among those who sought healthcare, 50% reported an out of pocket payment for healthcare
Do Chronic Diseases and Availability of Medications Predict Post-traumatic Stress Disorder (PTSD) among Syrian refugees in Jordan? Al-Samdi et al. [34]	Cross-sectional survey of adults SR (*n* = 765) (> 18 yrs) attending Caritas health centers in 6 cities in Jordan	N/A	71.9% did not receive sufficient medication
The Perceived Barriers of Health Care Among a Group of Non-camp Syrian Refugees in Jordan Ay et al. [35]	Cross-sectional survey of SR (*n* = 196), all age groups in non-camp setting Jordan	N/A	40.4% used PHC, 33.7% public governments, 14.6% private and 4.5 NGOs. Barriers: most frequent is cost followed by structural. Of cost, the cost of transportation was most frequent.63.6% reported an out-of-pocket expenditure for medical service.
Health status and health needs of older refugees from Syria in Lebanon Strong et al. [36]	Cross-sectional survey of older adults SR (*n* = 167) (> 60 yrs) attending Caritas clinics in 5 cities in Lebanon	HT (53%), DM (38%), CVD (28), high cholesterol (22%), lung disease (11%).	Cost was the main carrier to healthcare (87%).
Prevalence, care-seeking, and health service utilization for non-communicable diseases among Syrian refugees and host communities in Lebanon Doocy et al. [37]	Cross-sectional survey of SR (*n* = 2062 HH), all ages in camps and outside camps and Host community in Lebanon	Over half (50.4%) of refugee and HC (60.2%) reported a member with NCD. Among adults: SR (HT: 7.4%, COPD: 3.8%; CVD 3.3% and DM: 3.3%). HC (HT: 10.7%, DM 6.3%, CVD 5.3%, COPD: 2.6%)	Refugees utilized (PHCC) (57.7%) most often while HC most in private clinics (62.4%). Overall, 69.7% of refugees and 82.7% of HC members reported an out-o f pocket consultation payment.
Pilot Testing and Implementation of a mHealth tool for Non-communicable Disease in a Humanitarian Setting Doocy et al. [38]	Intervention study on SR and HC (*n* = 793) ≥40 years or ≥ 18 with HTN or DM in 10 PHC in Lebanon	(Pilot study)	mhealth as effective tool to improve adherence to guidelines and quality of care
Guidelines and mHealth to improve Quality of Hypertension and Type 2 Diabetes Care for Vulnerable Populations in Lebanon: Longitudinal Cohort Sudy Doocy et al. [39]	Intervention study on SR and HC (*n* = 793) ≥40 years or ≥ 18 with HTN or DM in 10 PHC in Lebanon		mhealth as effective tool to improve adherence to guidelines and quality of care
Outcomes of coronary artery bypass surgery in Syrian refugees Demir et al. [40]	*Chart review* of SR (age = 58 ± 9.23 years) underwent coronary artery bypass surgery (*n* = 53) in Turkey		In light of the outcomes of the surgery, the authors recommended that patients should receive therapy for chronic disorders in addition to emergency interventions.
An overview of the health status of Syrian refugee children in a tertiary hospital in Turkey Bucak et al. [41]	*Chart review* of SR children (*n* = 104) in Adiyaman hospital, Turkey	T1DM (1%). Chronic malnutrition (using anthropometry) (20%). Anemia (blood results) (50%)	
A refugee camp in the center of Europe: clinical charactersitics of asylum seekers arriving in Brussels Van Berlaer et al. [42]	*Chart review* Cross-sectional of Asylum seekers (Syrian, Iraqis, Afghanis and Palestinians), (*n* = 3907) field hospital in Brussels, Belgium [All age groups]	More than 7% of patients reported comorbidities (*n* = 279), most commonly arterial hypertension (*n* = 103) and/or diabetes (*n* = 96). Patients also reported asthma (*n* = 19)	
Experience with migrants on Balkan Route from the Field Hospital on the Slovenian-Croatian Border Bydzovsky et al. [43]	*Chart review* Cross-sectional of Asylum seekers (Syrian, Iraqis, Afghanis and Palestinians) (*n* = 6142) field hospital in Dobova on the Slovenian Croatian borders [All age groups]	CVD: 11.67%, HT: 22%, COPD: 10%, DM 4.8%.	
On the ferries: the unmet health care needs of transiting refugees in Greece Shortall et al. [44]	*Chart review* of Refugees (Syria, Afghanistan and Iraq) (*n* = 1405) in Greece [All age groups]	39.4% of diseases were classified as NCD.	
Cancer awareness and Barriers to Seeking Medical Help Among Syrian Refugees in Jordan: a Baseline Study Al Qadire et al. [45]	Cross-sectional survey of SR adult (18–47 years), non-camp setting (*n* = 240), recruited from healthcare facilities in Jordan		Most common barrier to seeking healthcare is ‘no medical insurance (83.4%).
Cardiovascular disease risk and prevention among Syrian refugees: mixed methods study of Medecins Sans Frontieres programme in Jordan Collins et al. [46]	*Chart review* Cross-sectional of SR (ages < 18 and < 40) (*n* = 2907), recruited from 2 outpatient NCD clinics in Jordan	CVD: 20%, DM: 52%, high waist circumference: 73%	Only 23% had a documented WHO/ISH risk score documented of which 35% were *incorrec*t. 20% of subjects who were eligible were *not prescribed* lipid lowering medications. Healthcare professionals in the clinics perceived that individual health education sessions were often co-opted by more immediate medication needs
A Preliminary Description of Medical Complaints and Medication Consumption among 375 Syrian Refugees Residing in North Jordan Gammoh [47]	*Chart review* Cross-sectional of SR Adults (> 20 yrs) at PHC (*n* = 220) in Jordan	CVD: 28%; HT: 25%, Respiratory diseases: 7%	N/A

^a^*SR* Syrian refugee, *HH* Households, *HT* hypertension, *DM* Type 2 Diabetes Mellitus, *T1DM* Type 1 Diabetes Mellitus, *N/A* not applicable

^b^In this table, only original articles were described

#### Prevalence of NCDs among Syrian refugees

Regarding the prevalence of NCDs among adult Syrian refugees, two cross-sectional, population-based surveys were conducted, one in Lebanon (*n* = 2062 households) and the second in Jordan (*n* = 1550 households). Both studies aimed at estimating the prevalence of NCDs among Syrian refugees living in a non-camp setting and found that almost one in two households had a member with NCDs in both Lebanon and Jordan [29, 37]. These surveys also show that hypertension was the most common NCD among adult Syrian refugees (10.7% in Jordan and 7.4% in Lebanon), followed by Type 2 Diabetes Mellitus (T2DM) (6.1% in Jordan and 3.3% T2DM). In Lebanon, the survey by Doocy et al. included host community in addition to Syrian refugees’ households and showed a higher proportion of households having at least one adult with NCDs in the host community (60.2% vs 50.4%, respectively) [37]. This study also reported a higher prevalence of hypertension and T2DM among host community as compared to Syrian refugees (hypertension: 10.7%, T2DM: 6.3%). The prevalence of NCDs among older adults Syrian refugees were higher, with hypertension also most commonly reported (53.%), followed by T2DM (38.0%) [36]. Al Fahoum et al., in their study of the provision of health services in Jordan to Syrian refugees, reported higher rates of hypertension among Syrian Refugees living in camp settings in Jordan (41.0% among males and 30.0% among women) [29].

Six studies reported on the rate of NCDs, using chart review [41–44, 46, 47], two of which reported the rates among Syrian refugees only while others included other refugee populations such as Iraqi or Afghani. The results of these two studies showed that among NCDs, the most frequent diagnosis was CVD, followed by T2DM and hypertension [46, 47].

#### Healthcare needs of NCD among Syrian refugees

As for healthcare needs for NCDs’ patients, the studies which reported on the ‘sufficiency’ of healthcare were conducted in Jordan and showed that, of each four Syrian refugees, three reported insufficient healthcare [29, 32, 34]. Syrian refugees predominantly seek healthcare in primary healthcare centers. In Jordan, Doocy et al. (2016) and Ay et al. (2016) showed that 51.5 and 40.4% of survey respondents sought medical care from primary healthcare centers, respectively [31, 35]. In Lebanon, this rate was 57.7% among Syrian refugees [37], however the host community in the country, primarily accessed medical care services in private clinics (63.0%) [37].

Five studies addressed the barriers to seeking healthcare [30, 31, 35, 36, 45]. In all of these studies, cost or lack of medical insurance reported as the main barriers to seeking healthcare. Other barriers reported included lack of knowledge on where to seek healthcare, transportation costs and lack of trust in healthcare providers [30]. Four studies reported on prevalence of out-of-pocket expenditures for medical care among Syrian refugees. Their results indicated that more than one in two Syrian refugees pays out of pocket for his medical care [31, 33, 35, 37].

Only a few studies addressed the quality of care delivered to Syrian refugees. From the user perspective, 46.0% of Syrian refugees reported receiving ‘bad’ healthcare [29] and indicated that lack of trust in the quality of care was a main barrier to seeking medical care [30]. Using chart review, Collin et al. found that only 23.0% of patients presenting to the Medecins Sans Frontiers clinics had a documented WHO/International society of Hypertension risk score of who 35.0% were incorrect. In addition, their results indicated that 20.0% of subjects who were eligible were not prescribed lipid lowering medications. [46]. Also using chart review, Demir et al. recommended that therapy for chronic disorders should be delivered to Syrian Refugee patients undergoing coronary artery bypass surgery, in addition to surgical emergency interventions [40].

**Fig. 3:**
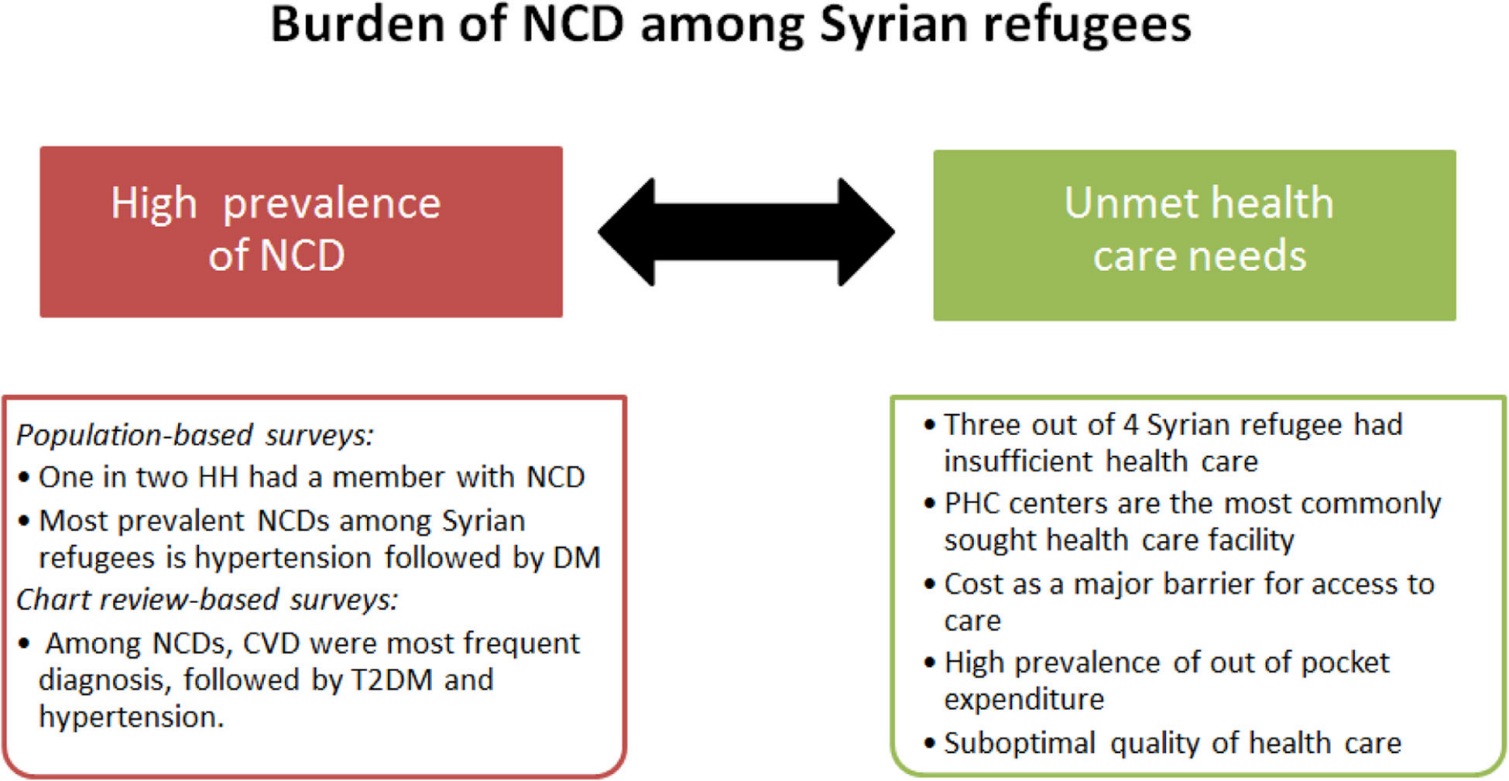
Summary of main findings of studies addressing NCDs among Syrian refugees

Of the studies reviewed, two described an intervention aimed to improve the quality of care of NCDs among Syrian refugees and the host community in Lebanon [38, 39]. Both studies evaluated the effect of using the mHealth app on quality of care and health outcomes in primary care settings and concluded that mhealth has the potential to improve adherence to guidelines and enhance the quality of care. The main findings regarding the prevalence of NCDs among Syrian refugees and their healthcare needs described earlier are summarized in Fig. 3.

## Discussion

The escalating rates of NCDs in Syria and its neighboring countries coupled with the protracted nature of the Syrian war led to a high burden of NCDs among the Syrian crisis-affected populations in Iraq, Jordan, Lebanon, and Turkey. A review of NCDs among urban refugees and asylum-seekers in developing countries called for the recognition that NCDs represent a new challenge in refugee operations and that NCDs’ health care is often neglected [49]. Efforts by governments, humanitarian organizations and international agencies to effectively address this high burden of NCDs were hampered by a constellation of factors including lack of information on unmet population need; little consensus among humanitarian actors regarding an effective health care interventions; and no clear approach for prioritizing public health interventions, in addition to conflict-related insecurity and disruption to infrastructure, hampering continuity of chronic illness care [50].

The studies examined in this review tackled NCDs’ burden among Syrian refugees and addressed the four major types of NCDs (CVD, diabetes, cancer and COPD). The majority of these studies were descriptive and only two studies addressed the effectiveness of interventions. Such a limited number of studies on interventions is not in line with the recurrent calls urging a better understanding of effectiveness of interventions tackling NCDs among Syrian refugees [49, 51, 52]. It is noteworthy that none of the interventions described in these two studies aimed at preventing NCDs, rather they were focused on the management and treatment side of the diseases. The absence of studies of interventions aiming to prevent NCDs among Syrian refugees contradicts global efforts geared towards the prevention of these diseases, especially in crisis and fragile settings [53].

Furthermore, of the 34 reviewed papers, seven addressed the host communities and their challenges and only one research article, conducted in Lebanon, included subjects from the host community [37]. This finding is alarming in light of the high burden of NCDs in most of the host communities and the escalating tensions between these communities and Syrian refugees. In Lebanon and Jordan, Syrian refugees usually reside in poor communities and share their limited resources. This situation led in many instances to pressures on the health systems and eventually less access to basic healthcare services for vulnerable host communities. The perception that Syrian refugees get preferential treatment, perhaps due to humanitarian assistance and interventions directly targeting them, has been reported to exacerbate the friction between host communities and Syrian refugees [54]. Addressing the health care needs of the host community will lessen the existing tension with the Syrian refugees and aid in the integration of the latter in the host country, which in turn may enhance access of the refugees to available health care services. Therefore, it is recommended that future research on alleviating the burden of NCDs among Syrian refugees also include the host communities. From a bibliometric perspective, this review showed an increasing interest among members of the scientific community to address NCDs among Syrian refugees, as evidenced by the number of published documents per year since the start of the Syrian war in 2011. This momentum of research productivity reflects an opportunity that could contribute to the generation of needed evidence to effectively address NCDs among Syrian refugees, if directed to fill in the existing knowledge gaps. Among these gaps, as reported by a systematic review of the effectiveness of interventions for NCDs in humanitarian crises are a better understanding of NCDs’ delivery models in fragile settings; using comparison groups (where appropriate); examining the costs and sustainability of interventions; and addressing the bias in settings where standard randomized control studies are not feasible [22].

In this review, the synthesis of results of original articles addressing NCDs among Syrian refugees led to two main findings that warrant further discussion: a high prevalence of NCDs among Syrian refugees and unmet healthcare needs. It is important to note however that these findings ought to be considered with caution due to the limited number of studies and the heterogeneity of their results. This review showed that one in two Syrian refugee households had a member with NCDs. Such a significant prevalence was also noted by the Disaster and Emergency Management Survey, which showed that 10.0% of Syrian refugees reported a form of NCDs [55]. This finding adds further evidence to existing knowledge that refugees from middle income countries such as Syria present a different demographic and disease profile burden than the classical profile of refugees fleeing conflicts in Africa where diseases of poverty (such as diarrhea, cholera, or malaria) are more prevalent [56]. Examination of reported prevalence rates of specific NCDs among Syrian refugees showed that the most commonly reported NCD was hypertension (7.4–9.7%), followed by T2DM (3.3–5.3%). This findings is also in line with the results of the Disaster and Emergency Management Survey which showed that hypertension and diabetes were the most common NCDs among Syrian refugees [57]. It is important to note that the prevalence rates reported for hypertension and diabetes among Syrian refugees in studies included in this review are lower than those previously estimated in Lebanon (28.8 and 14.9% for hypertension and diabetes, respectively) and Syria (29.5 and 8.8% for hypertension and diabetes, respectively) [37, 58–60]. Such discrepancies could be explained by the fact that the prevalence studies included in this review were based on self-reporting of diseases, in which case undiagnosed cases and cases that poorly understood their condition were missed [37]. Furthermore, Doocy et al. argued that selective migration of healthier Syrians could be a reason for the lower estimates for hypertension and diabetes prevalence among Syrian refugees [37].

This review highlighted insufficient healthcare for NCDs’ patients among Syrian refugees. The massive influx of Syrian refugees to neighboring countries has put enormous pressures on their already fragile health systems (especially in Lebanon and Jordan). In this situation, these health systems struggled to cope with the acute healthcare needs and often neglected the treatment and management for NCDs resulting in disruption of care, reduced functional capacity, disease exacerbation, worsening of disease prognosis, or even death [3, 61]. Such a situation comes along despite global calls for a systematic integration of affordable NCDs’ treatment in the health system. For instance, complementary to, and building on, the World Health Organization (WHO) Global Action Plan for the Prevention and Control of Non-communicable Diseases – 2013-2020, the following statement appeared in the Sendai Framework for Disaster Risk Reduction 2015–2030 (Sendai Framework): “*people with life-threatening and chronic disease, due to their particular needs, should be included in the design of policies and plans to manage their risks before, during, and after disasters, including having access to life-saving services”*. This statement underlines the need to include NCDs’ care as an integral part of policy design and implementation of plans to manage risks among Syrian refugees, including having sufficient access to healthcare [62, 63]. Despite such a high-level recognition of the importance of integrating NCDs’ care and management in humanitarian setting, actions on the ground are usually impeded by significant knowledge gaps in effective strategies and policies.

In all reviewed papers in this study, primary healthcare centers in host countries were found to be at the forefront of healthcare delivery for NCDs among Syrian refugees. This finding is in accordance with previous literature citing the national health systems particularly primary healthcare centers as the first line of defense against health crises [64]. The important role which primary healthcare play in healthcare delivery among Syrian refugees underscores the need for strengthening the resilience of existing health systems in host countries, especially primary healthcare, in order to be able to maintain and deliver their routine functions while withstanding the overwhelming health needs of refugees [65]. In their proposal for a resilience index, E Kruk et al. stated that resilience requires planning and investment at all levels including system-related issues such as human resources for health and health information systems as well as healthcare delivery components such as isolation wards, protective equipment and surveillance. The authors also emphasized the importance of building collaboration and trust with communities ahead and amid crises [64].

In this review, the main barrier to sufficient healthcare for NCDs among Syrian refugees was related to healthcare costs or the absence of medical insurance. This finding is in line with previous studies that showed that cost is the main barrier for refugees seeking healthcare for NCD [66, 67]. In the context of Syrian refugees residing in either Lebanon or Jordan, although primary healthcare (which were found in this review to be the most commonly sought healthcare facility) are predominantly free of charge, the recent cuts in humanitarian assistance led many Syrian refugees to assign a lower priority for healthcare spending [30]. In this context, it remains important to note that the treatment for NCDs, in many instances, requires more than primary care, especially in advanced stages of the diseases. Furthermore, Ay et al. argued that the cost of medication could become a barrier especially when refugees had to buy medicines in the case of a shortage or discontinuation of declared-free drugs [68]. Another finding of concern in this review was the suboptimal quality of care for NCDs among Syrian refugees, whether assessed from the perspectives of the users or providers. This finding could be due to the fact that many public healthcare facilities in neighboring host countries such as Lebanon or Jordan could be lacking the trained expertise and/or are overwhelmed by the demands on their already weak infrastructures [69].

The findings of this review ought to be considered in light of a couple of limitations. First, the search for documents was limited to those available electronically and could have missed relevant information not archived in this format. The search employed in this review however was comprehensive and thus provided a clear depiction of overall NCDs’ burden among Syrian refugees and their host communities. Second, similar to other scoping reviews, reviewed documents were not screened for quality and, thus, have large variations in study methodologies and sampling.

## Conclusions

Current health policies and interventions among Syrian refugees seem to be lagging behind the profound global changes in humanitarian crises. Mainly, these crises became more protracted in nature with infectious diseases and malnutrition being replaced with longer term health conditions such as NCDs. Findings from this review highlight two main issues related to NCD among Syrian refugees: first a significant prevalence of NCDs and second the largely unmet healthcare needs of Syrian refugees. As a result, this review underscores the call for a better understanding of NCDs and interventions in humanitarian crises. More specifically, future research on NCDs among Syrian refugees ought to consider the following issues:

1. Focus on identifying quality interventions: The focus should be on intervention-based research, in addition to cross-sectional survey based investigations, to better inform policies and properly guide humanitarian assistance. In addition, interventions aiming to improve standards and quality of NCDs’ care are needed.
2. Consider the full intervention spectrum: Attention should be given to the prevention of NCDs in addition to their management and treatment
3. Include all relevant populations: The host communities should be included in NCDs’ programs and interventions
4. Strengthen service delivery: Future research should examine strategies to enhance the resilience of primary healthcare centers in host countries (especially Lebanon and Jordan), given their strategic role in healthcare delivery for Syrian refugees. Such strategies could address the centers’ medicines management capacity, health workforce, infrastructure and financing systems, as well as leadership & governance capacity [70]

Although solutions to alleviate the burden of NCDs among Syrian may seem as an intractable task, the impressive progress that has been made in the management of communicable diseases clearly illustrates that large-scale change could be feasible with global collaboration, research and advocacy. In fact, the research momentum for NCDs’ prevention and management found in this review presented an opportunity, which if geared towards feasible and effective interventions, could lead to improved humanitarian national and international actions to prevent, control and ultimately reduce the burden of NCDs.

## Additional files

Additional file 1: Data Extraction Form. (DOCX 26 kb)

Additional file 2: Characteristics of original studies included in the scoping review (*n* = 19)*. (DOCX 38 kb)

## Abbreviations

COPD: Chronic obstructive pulmonary disease
CVD: Cardio-vascular diseases
INGO: International non-governmental organization
LMIC: Low and middle income countries
NCD: Non-communicable diseases
NGO: Non-governmental organization
T2DM: Type 2 Diabetes Mellitus
