# Supplementary material for: Burden of non-communicable diseases among Syrian refugees: a scoping review
Source: BMC Public Health. 2019 May 24;19:637. doi: 10.1186/s12889-019-6977-9 (PMC6534897; doi:10.1186/s12889-019-6977-9)
Supplement: Supplementary file 1 — Data Extraction Form. (DOCX 26 kb) [file 12889_2019_6977_MOESM1_ESM.docx]

# Additional file 1: Data Extraction Form

1. **General information about the article**
   1. Code Name (person who enters the data)
   2. Title of the study (string) (copy from article)
   3. Language of publication
      - 1=English
      - 2=Arabic
   4. DOI ofthe journal article (if applicable)
   5. Document type
      - 1=Journal Article
      - 2=Book chapter
      - 3=Short paper
      - 4=Report
      - 5=Case study
   6. Journal Name (full)
   7. Year of publication of the document type (date of print)
   8. Journal IF current (if applicable)
   9. Affiliation of ALL the authors (select all that apply 1=Yes and 2=No)
      1. Academic institutions
      2. NGO
      3. Governmental agencies
      4. Other
      5. Unknown
   10. Specify other (string)
   11. Affiliation of the **corresponding** author (only the name of the institution the author works in) (string)
   12. Name of the **corresponding author** (written as family name then the first letter of the first name and middle name only if provided in the paper e.g.: Sibai AM, Haida M, Fouad MF
   13. Country of the affiliating institution of the **corresonding author** (string)
   14. Affiliation of the **first author** (only the name of the institution the author works in) (string)
   15. Name of the **first author** (written as family name then the first letter of the first name and middle name only if provided in the paper e.g.: Sibai AM, Haida M, Fouad MF
   16. Country of the affiliating institution of the **first author** (string)
   17. Study Design
       - 1=Case report
       - 2=Case series
       - 3=Cross-sectional study
       - 4=Case-control study
       - 5=Cohort study
       - 6=RCT
       - 7=Systematic Review/Meta analysis
       - 8=Review (Literature review)
       - 9=Focus Group Discussions/KEY Informant Interviews
       - 10= Ethnographic studies
       - 11=Laboratory/Basic Science studies/Basic Science (animal-based/pathological/invivo studies) [RCT phase 0]
       - 12=N/A
2. **Study population (Who)**
   1. Sample size (numeric) [include the total sample in case the study had two subsamples, i.e. women and children]
   2. Sampling procedure

1= Probability sampling technique [simple random sampling, multi-stage cluster random sampling, proportionate sampling, …etc…]

2= Non-probablity sampling [quota sampling, convenient sampling, snowballing, respondent-driven sampling].

- 1. Data collection mode (select all that apply) (1=Yes and 2=No)
     1. Face to face household/site survey
     2. Telephone landline interview
     3. Telephone cellphone interview
     4. Telephone dual-mode interview
     5. Chart/Record extraction
     6. Online survey
     7. N/A
  2. Sample includes Syrian refugees only (1=Yes and 2=No)
  3. Sample includes Other refugees (1=Yes and 2=No)
  4. If yes , specify other refugees (string)
  5. Sample includes Syrian refugees and IDPs in Syria (1=Yes and 2=No)
  6. Sample includes Host communities? (1=Yes and 2=No)
  7. Sex of Syrian refugees
     - 1= Males only sample
     - 2=Females only sample
     - 3=Both males and females included in the sample
     - 4=N/A
  8. Composition of Syrian refugees sample (more than one answer can apply) (1=Yes and 2=No)
     1. Pregnant and breast feeding
     2. Infant and child (<2)
     3. Children(2<9 years)
     4. Children and Adolescents (> 10 and <18 years)
     5. Young adults are between 18-25
     6. Adults (above 18)
     7. Older population (above 50)
     8. N/A

1. **Study location (Where)**
   1. Host country of the Syrian refugees (more than one answer could be selected) (1=Yes and 2=No)
      1. Lebanon
      2. Jordan
      3. Turkey
      4. Greece
      5. Italy
      6. USA
      7. Australia
      8. Germany
      9. Sweden
      10. France
      11. Britain
      12. Canada
      13. Egypt
      14. Iraq
      15. Austria
      16. Switzerland
      17. Poland
      18. Netherlands
      19. Belgium
      20. Other
      21. Other host country specify (string).
   2. Refugees in the study are living in (more than one answer could be selected) (1=Yes and 2=No)
      1. Informal tented settlement(s)
      2. Formal tented settlement(s)/Initial reception centers/Collective accomodation centers
      3. Decentralized accommodation
      4. Not specified
      5. Other
      6. Specify other (string)
   3. Study sample (subjects) recruited from: (more than one answer could be selected) (1=Yes and 2=No)
      1. Healthcare facility (Hospital, PHC)
      2. Refugee Camps
      3. Schools
      4. NGO facility
      5. Community (population-based)
      6. Other
      7. N/A (reviews)
      8. Specify of other (string)
2. **Study characteristics**
   1. Type of data collected (Select all that apply) (1=Yes and 2=No)
      1. Qualitative (go to E)
      2. Quantitative (go to F)
      3. Mixed methods (go to E)
   2. Start Year of the study (i.e. 2012) (numeric)
   3. Specify the name(s) of the Funding organization (string)
   4. Did the author(s) report conflict of interests (1=Yes and 2=No)
   5. Was the study IRB approved? (1=Yes 2=IRB was 3=not sought 4= Not mentioned)
   6. Response rate/Dropout rate (numeric)
3. **In case the data collected was qualitative**
   1. Type of qualitative study (select all that apply) (1=Yes and 2=No)
      1. Focus group discussions
      2. Key informant interviews
      3. Focus group discussions and key informant interviews
      4. Other, specify (string)
   2. Number of focus group discussions/key infromant interviews (numeric) (insert actual number)
   3. For focus group discussions, how many per focus group? (numeric)
   4. Were the focus group discussions/key informant interveiws taped (1=Yes and 2=No)
   5. Languge of the focus group discussions/key informant interviews (string)
   6. How was the thematic analysis conducted?
      - 1=Manual
      - 2=Using INVIVO
      - 3=Other computer software, specfiy (string)
   7. How was the thematic analysis conducted? Other specify
4. **In case the study was an intervention**
   1. Aim of the intervention (string) (pull from the abstract)
   2. Description of intervention (string) (pull from abstract)
   3. Type of intervention
      - 1=Randomized control trials
      - 2=Control trials
      - 3= Randomized trials
      - 4= Community-based intervention
      - 5=Other
   4. Specify Other interventions (string)
   5. Eligibility criteria for participants (string) (pull from abstract)
   6. Does the intervention have a control/comparison group? (1=Yes and 2=No)
   7. Setting and location where intervention was conducted (string) (pull from abstract)
   8. Primary and secondary outcome measures (string) (pull from abstract)
   9. Type of evaluation for the intervention
      1. Type of assessment (1=Outcome evaluation and 2=Process evaluation)
      2. Pre-assessment (1=Yes and 2=No)
      3. Post-assessment (1=Yes and 2=No)
   10. Main results (string) (pull from the abstract)
   11. Total Duration of intervention (in weeks) (1 month = 4 weeks) (numeric)
   12. Number of sessions/contact with sample (numeric)
   13. Cost of intervention (1=Not specified and 2=specified)
   14. If cost specified, indicate how much (in US dollars) (numeric)
   15. Was cost-efficiency assessed? (1=Yes and 2=No)
   16. If yes, specify how (string)
   17. Limitations of the intervention (string) (pull from abstract)
5. **Type of NCDs**
   1. CVD & Stroke (1=Yes and 2=No)
   2. Diabetes (1=Yes and 2=No)
   3. Cancer (1=Yes and 2=No)
   4. Type of Cancer
      - 1=Brest
      - 2=Gastric
      - 3=Colon
      - 4=Prostate
      - 5=Lung
      - 6=Lukemia
      - 7=Other (add string and specify)
   5. Other type of Cancers specify (string)
   6. Palliative care (1=Yes and 2=No)
   7. Chronic lung dysfunction/Asthma/COPD (1=Yes and 2=No)
   8. Renal dysfunction due to CVD (1=Yes and 2=No)
   9. Chronic diseases/NCD in general (1=Yes and 2=No)
   10. Other Outcomes (1=Yes and 2=No)
   11. Specify other outcomes (string)
6. **Risk factors addressed**
   1. Genetic predisposition (1=Yes and 2=No)
   2. Family history (1=Yes and 2=No)
   3. Family/parenting environment (1=Yes and 2=No)
   4. Tobacco (1=Yes and 2=No)
   5. Nutrition/ diet (1=Yes and 2=No)
   6. Alcohol and Other Substance Abuse (1=Yes and 2=No)
   7. Physical Inactivity (1=Yes and 2=No)
   8. Hypertension (1=Yes and 2=No)
   9. High blood cholesterol or hyperlipidemia (1=Yes and 2=No)
   10. High blood glucose (1=Yes and 2=No)
   11. Metabolic Syndrome (1=Yes and 2=No)
   12. Obesity/Overweight (1=Yes and 2=No)
   13. Social determinants (including Gender, Education, Levels of income, Urban/rural, socioeconomic status) (1=Yes and 2=No)
   14. Structural Factors/ Access to care/cost (1=Yes and 2=No)
   15. Other risk factors (1=Yes and 2=No)
   16. Specify other risk factors (string)
7. **Theme in relation to NCD**
   1. Genetics (1=Yes and 2=No)
   2. Family History (1=Yes and 2=No)
   3. Diagnosis (more medical viewed paper) (1=Yes and 2=No)
   4. Prevention and Control (screening evaluations, intervention studies) (1=Yes and 2=No)
   5. Management and Complications (intervention studies) (1=Yes and 2=No)
   6. Policy/ health system/ insurance/ pension plans (1=Yes and 2=No)
   7. Etiology/risk factoriology/determinants/analytical (1=Yes and 2=No)
   8. Prevalence/Incidence/distribution/descriptive (1=Yes and 2=No)
   9. Was a theory of behavioral change used (1=Yes, 2=No and 3=N/A)
   10. If yes, were the constructs of the therory of behavioral change assessed in the intervention? (1=Yes and 2=No)
8. **General variables**
   1. Hyperlink (Copy it as it is)
   2. DOI (Copy it as it is)
