# Supplementary material for: Burden of non-communicable diseases among Syrian refugees: a scoping review
Source: BMC Public Health. 2019 May 24;19:637. doi: 10.1186/s12889-019-6977-9 (PMC6534897; doi:10.1186/s12889-019-6977-9)
Supplement: Supplementary file 2 — Characteristics of original studies included in the scoping review (n = 19)*. (DOCX 38 kb) [file 12889_2019_6977_MOESM2_ESM.docx]

# Additional file 2: Characteristics of original studies included in the scoping review (n=19)^*^.

| Author/year | Title | Year of study/data | Host country | Aim/Description | Study design | Study population | Inclusion criteria | | NCD(s) reported | Main Findings |
| --- | --- | --- | --- | --- | --- | --- | --- | --- | --- | --- |
| Gammouh et al. 2015 [36] | Chronic Diseases, Lack of Medications, and Depression Among Syrian Refugees in Jordan, 2013–2014 | 2013 | Jordan | To analyzed participants’ demographics, depression, previously diagnosed chronic diseases, and newly diagnosed chronic diseases and the availability of medications | Cross-sectional | Syrian refugees only  n=765  18+ years | Syrian refugee living in Jordan during the study data collection period, aged 18 years or older, and willing to participate in the study | | Chronic disease | Of 765 refugees who participated, about one-third demonstrated significant depression. Descriptive analyses showed that depression was comorbid in 35% of participants with previously diagnosed chronic diseases and in 40% of participants with newly diagnosed chronic diseases. Newly diagnosed chronic diseases and lack of medications significantly contributed to depression, but the regression model as a whole explained less than 5% of the variance. |
| Mechili et al. 2015[27] | The Provision of Health Services in Jordan to Syrian Refugees | 2013 | Jordan | The purpose of our study is to explore refugees' attitudes towards health services (i.e. evaluation of the adequacy of healthcare services and the degree of their satisfaction by the services provided). | Cross-sectional | Syrian refugees only  n=120  ≥18 years | Syrian refugees in Zaatary camp older than 18 | | Health problems such as asthma, hypertension | Approximately 75% of people argued that they received insufficient healthcare services, with a 43% of people predicted that their health condition in future will significantly exacerbate. The probability of someone with poor health status to report an inadequate health service highly increases by 41.1 percentage points compared to someone that have a good health status. A strong association was found between a negative evaluation of health services and future projections of health status. |
| Doocy et al. 2015[40] | Prevalence and care-seeking for chronic diseases among Syrian refugees in Jordan | 2014 | Jordan | To characterize prevalence of NCDs and better understand issues related to NCD care seeking. | Cross-sectional | Syrian refugees only  n=1550 households  All age groups | Only Syrian households arriving in Jordan in 2011 or after were eligible to participate in the survey | | Hypertension, cardiovascular disease, diabetes, chronic respiratory disease and arthritis | Among adults, HTN prevalence was the highest (9.7 %), arthritis (6.8 %), DM (5.3 %) chronic respiratory diseases (3.1 %) and CVD (3.7 %) Of the 1363 NCD cases, 84.7 % received care in Jordan; of the five NCDs assessed, arthritis cases had the lowest rates of care seeking at 65 %, Individuals from households in which the head completed post-secondary and primary education, respectively, had 89 % and 88 % lower odds of seeking care than those with no education (p = 0.028 and p = 0.037, respectively). |
| Doocy et al. 2016[38] | Health service access and utilization among Syrian refugees in Jordan | 2014 | Jordan | To characterize health seeking behaviors and issues related to accessing care. | Cross-sectional | Syrian refugees only  n=1550 households  Adults | Only adults in Syrian households arriving in Jordan in 2011 or after were eligible to participate in the survey | | NCDs in general | Care-seeking was high with 86.1 % of households reporting an adult sought medical care the last time it was needed. Approximately half (51.5 %) of services were sought from public sector facilities, 38.7 % in private facilities, and 9.8 % in charity/NGO facilities. Among adult care seekers, 87.4 % were prescribed medication during the most recent visit, 89.8 % of which obtained the medication. Overall, 51.8 % of households reported out-of pocket expenditures for the consultation or medications at the most recent visit (mean US$39.9, median 4.2 USA$ |
| Gammouh 2016[35] | A Preliminary Description of Medical Complaints and Medication Consumption among 375 Syrian Refugees Residing in North Jordan | 2013 | Jordan | This study aimed to describe medical complaints and medication consumption in a Syrian refugee population residing in north Jordan | Cross-sectional | Syrian refugees only  n=375  All age groups | Syrian refugees who attended a primary care center organized for charity purposes for three days by the ACCTS (3 days free) | |  | Almost 30% of the study sample had pain, infectious diseases and chronic diseases. A total of 280 prescriptions were issued, consisting mainly of anti-infective, anti-inflammatory and anti-hypertensive drugs. |
| Doocy et al. 2016[42] | Health Service Utilization among Syrian Refugees with Chronic Health Conditions in Jordan | 2014 | Jordan | To characterize health seeking behaviors and issues related to accessing care for hypertension, diabetes, cardiovascular diseases, chronic respiratory diseases, and arthritis | Cross-sectional | Syrian refugees only  n=1363  households  All age groups | Only Syrian households arriving in Jordan in 2011 or after were eligible to participate in the survey | | Hypertension, cardiovascular disease, diabetes, chronic respiratory disease and arthritis | Of 1363 cases with a chronic health condition diagnosis, 84.7% had received care in Jordan. Public facilities faced a heavy burden serving over half (53.9%) of care-seekers; the remainder received care in the private (29.6%) and NGO/charity (16.6%) sectors. Individuals with non-communicable diseases (NCDs) in the central region of Jordan and with arthritis had the lowest rates of care-seeking when compared to other regions and conditions. Overall, 31.6%of care-seekers had an out-of-pocket payment for the most recent care seeking event which averaged 18.8 USD excluding medication |
| Al-Samadi et al.2016[37] | Do Chronic Diseases and Availability of Medications Predict Post-traumatic Stress Disorder (PTSD) among Syrian Refugees in Jordan? | 2013 | Jordan | The primary objective of this study was to examine if previous chronic diseases, newly diagnose chronic diseases in Jordan and medication shortage can predict PTSD in cohort Syrian residing in Jordan. | Cross-sectional | Syrian refugees only  n=765  18+ years | Adults Syrian refugees, aged greater than 18 and older and to be resident in Jordan as refugees for at least the last 6 months | | Chronic disease | Majority (81%) had Lower PTSD scores, PTSD was comorbid with chronic disease in more than half of the participants, logistic regression analysis indicated that job status and availability of medication were predictors of PTSD |
| AY et al. 2016[39] | The Perceived Barriers of Access to Healthcare Among a Group of Non-camp Syrian Refugees in Jordan | 2014 | Jordan | The aims of this study were to identify the most needed healthcare services, accessibility of various healthcare services, and barriers to access as perceived by a group of Syrian refugees living in non-camp settings in Jordan and to compare accessibility among different groups | Cross-sectional | Syrian refugees only  n=196  All age groups | Syrian refugees living outside the camps throughout Jordan since January 2012 | | Chronic diseases | In addition to the prevalent acute and communicable diseases, chronic diseases and dental problems were common. Preventive and primary healthcare were more accessible than advanced services. Structural and financial barriers hindered access. The specific survey location and governorate were associated with a difference in reported access. Registration status, health provider, duration, and out-of-pocket payment did not affect accessibility. |
| Al Qadire et al. 2017[41] | Cancer Awareness and Barriers to Seeking Medical Help Among Syrian Refugees in Jordan: a Baseline Study | N/A | Jordan | To explore the level of cancer knowledge and barriers to seeking care among Syrian refugees in Jordan | Cross-sectional | Syrian Refugees only  n=241  18-47 years | The subjects are Syrian refugees living in the northern cities of Jordan. Participants must be older than 18, not living in a camp, able to read and write, physically able to complete the questionnaire and willing to take part in the study. Otherwise, participants were excluded | | Cancer | Participants were able to recognize a low number of symptoms (mean 4.4, SD 2.3) and risk factors (4.7 (out of 11), SD 1.9). The most commonly reported barrier was having no medical insurance (83.4%). Refugees’ knowledge of symptoms and risk factors was generally unsatisfactory. Barriers to seeking medical care were prevalent. |
| Collins et al. 2017[34] | Cardiovascular disease risk and prevention amongst Syrian refugees: mixed methods study of Médecins Sans Frontier’s programme in Jordan | N/A | Jordan | To evaluate MSF’s use of total CVD risk based prevention strategies among Syrian refugees in northern Jordan to identify opportunities to improve total CVD risk based guidance for humanitarian settings. | Cross-sectional | Syrian refugees and host community  n=2907  ≥40 years and ≥18 years with NCD risk factors | all patients aged ≥40 were eligible for inclusion, in addition to adults (≥18) under 40 who smoked, were diabetic, had a family history of CVD or diabetes in a first or second degree relative, or a high waist circumference | | CVD and risk factors (diabetes, smoking, high blood pressure, dyslipidemia, increased WC, family history of CVD or DM) | One-fifth (20.9%) of patients had a history of CVD while 56.8% of patients had a WHO/ISH risk of <10%. Only 23.3% of Pts had a documented WHO/ISH risk score of which 65% were correct. 60.4% of patients were eligible for lipid-lowering treatment and 48.3% of these patients were prescribed it. Analysis of interviews with sixteen MSF staff - had confusion about when and how to use the risk charts, tended to favor lifestyle intervention over drug treatment, and had uncertainty about the role of lipid-lowering treatment in primary but not secondary prevention. |
| Burnham et al. 2015[29] | Health status and health needs of older refugees from Syria in Lebanon | 2013 | Lebanon | This study sought to characterize the physical and emotional conditions, dietary habits, coping practices, and living conditions of this elderly population arriving in Lebanon between March 2011 and March 2013. | Cross-sectional | Syrian and Palestinian (from Syria) refugees  n=210  ≥60 years | A Syrian national or Palestinian refugee older than 60 years, resident in Syria who entered Lebanon after March 2011 was considered a refugee for the purposes of this study. | | Hypertension, diabetes, heart disease, high cholesterol | 2/3 described their health status as poor or very poor. Most reported at least 1 NCD, with 60% having HTN, 47% DM, and 30% indicating some form of heart disease. Difficulties in affording medicines were reported by 87%. Physical limitations were common: 47% reported difficulty walking and 24% reported vision loss. About 10% were physically unable to leave their homes and 4% were bedridden. Most required medical aids such as walking canes and eyeglasses. Diet was inadequate, regularly reducing portion sizes, skipping meals, and limiting intake of fruits, vegetables, and meat |
| Doocy et al. 2016[28] | Prevalence, care-seeking, and health service utilization for non-communicable diseases among Syrian refugees and host communities in Lebanon | 2014 | Lebanon | To assess the health status, unmet needs, and utilization of health services among Syrian refugees and host communities in Lebanon. | Cross-sectional | Syrian refugees and host community  n=2062  All age groups | Syrian households arriving to Lebanon after 2011, and Lebanese host community households | | Hypertension, cardiovascular disease, diabetes, chronic respiratory disease and arthritis | Over half (50.4 %) of refugee and HC (60.2 %) reported a member with one of the five NCDs. Care-seeking for NCDs among refugees and HC was high with 82.9 and 97.8 %, Refugees utilized (PHCC) (57.7 %) most often while HC most in private clinics (62.4 %). Overall, 69.7 % of refugees and 82.7 % of HC members reported an out-o f pocket consultation payment. |
| Doocy et al. 2017[45] | Pilot Testing and Implementation of a mHealth tool for Non-communicable Diseases in a Humanitarian Setting | 2015 | Lebanon | Its two research aims were (1) to develop, adapt, and test existing standards and guidelines for treatment, including counseling, of persons with hypertension and type 2 diabetes (or both) and (2) to evaluate the effectiveness of an mHealth tool | Intervention | Syrian refugees and host community  n=793  ≥40 years or ≥18 with HTN or DM | Lebanese and Syrian refugees attending one of the 10 healthcare centers supported by international organization for migration or the international medical corps, Individuals without a diagnosis of hypertension or type 2 diabetes, those aged less than 40 years, and adults lacking capacity to independently participate in interviews were excluded | | Diabetes, hypertension and BMI | Compared with baseline record extraction, recording of blood pressure (BP) readings (-11.4%, P<.001) and blood sugar measurements (-6.9%, P=.03) significantly decreased following the implementation of treatment guidelines. Recording of BP readings also decreased after the mHealth phase as compared with baseline (-8.4%, P=.001); |
| Doocy et al. 2017[44] | Guidelines and mHealth to Improve Quality of Hypertension and Type 2 Diabetes Care for Vulnerable Populations in Lebanon: Longitudinal Cohort Study | 2015 | Lebanon | Its two research aims were (1) to develop, adapt, and test existing standards and guidelines for treatment, including counseling, of persons with hypertension and type 2 diabetes (or both) and (2) to evaluate the effectiveness of an mHealth tool | Intervention | Syrian refugees and host community  n=793  ≥40 years or ≥18 with HTN or DM | Lebanese and Syrian refugees attending one of the 10 healthcare centers supported by international organization for migration or the international medical corps, Individuals without a diagnosis of hypertension or type 2 diabetes, those aged less than 40 years, and adults lacking capacity to independently participate in interviews were excluded | | Diabetes, hypertension and BMI | Compared with baseline record extraction, recording of blood pressure (BP) readings (-11.4%, P<.001) and blood sugar measurements (-6.9%, P=.03) significantly decreased following the implementation of treatment guidelines. Recording of BP readings also decreased after the mHealth phase as compared with baseline (-8.4%, P=.001). |
| Demir et al. 2016 [43] | Outcomes of coronary artery bypass surgery in syrian refugees | 2014 | Turkey | The aim of the present study was to present the outcomes of coronary artery bypass surgery in Syrian refugees. | Cross-sectional | Syrian refugees only  n=53  59±9.23 | Syrian refugees, who had undergone coronary artery bypass surgery between 2012-2014 at the Sanliurfa Mehmet Akif hospital | | Cardiovascular diseases, diabetes, COPD smoking | Of the patients, 18.9% sustained myocardial infarction, 34% had diabetes, 28.3% had COPD, and 52.8% were smokers. Two patients had emergency surgery and 51 patients had elective surgery. In the postoperative period, five patients (9.4%) were found to have atrial fibrillation. Cerebrovascular disease was observed at three patients (5.7%) in the postoperative period, and one patient had wound infection. A total of three patients (5.7%) died. |
| Bucak et al. 2017 [30] | An overview of the health status of Syrian refugee children in a tertiary hospital in Turkey | 2015 | Turkey | The purpose of this study was to evaluate the health status of Syrian refugee children presenting to a tertiary hospital in Turkey. | Cross-sectional | Syrian refugees only  n=104  0-18 years | The medical files of Syrian refugee children (0–18 years) presenting to our hospital pediatric clinic between 1 and 30 November 2015, | | Case with Diabetes | Examination of ICD codes revealed that only 9 (8.7%) had received routine pediatric examination diagnoses, the most common being acute infectious diseases. In the light of data from patients’ records, 87 (83.7%) patients had no chronic disease, while of the other 17 (16.3%); 7 (6.7%) were diagnosed with cerebral palsy, 5 (4.8%) with epilepsy, 4 (3.8%) with beta thalassemia major, and one patient each (1%) with diabetes mellitus, thalassemia carriage, and magnesium deficiency. |
| Van Berlaer et al. 2016 [31] | A refugee camp in the center of Europe: clinical characteristics of asylum seekers arriving in Brussels | 2015 | Belgium | This study aims to describe the demographic and clinical characteristics of asylum seekers who arrived in a huddled refugee camp, in the center of a well-developed country with all medical facilities. | Cross-sectional | Syrian, Iraq, Morocco, Afghanistan, Palestine refugees  n=3907  All age groups | All patients presenting spontaneously to the field hospital or examined by the outpatient assistance teams were eligible for inclusion. Patients with missing date of presentation, chief complaint or single primary diagnosis were excluded afterwards. | | Cases with hypertension, diabetes and asthma | Over 11% of patients suffered from injuries, but these were outnumbered by the proportion of patients with respiratory (36%), dental (9%), skin (9%) and digestive (8%) diagnoses. More than 49% had features of infections at the time of the consultation. More than 7% of patients reported comorbidities (n=279), most commonly arterial hypertension (n=103) and/or diabetes (n=96). Patients also reported asthma (n=19), old fractures (n=15), epilepsy (n=14), rheumatism (n=10), recent delivery. |
| Bydzovsky et al. 2016[32] | Experience with migrants on Balkan Route from the Field Hospital on the Slovenian-Croatian Border | 2015 | Slovenian-Croatian border | The aim of the study was to assess the spectrum of diseases in the population of migrants in 2015/2016 arriving to field hospital in Dobova on the Slovenian-Croatian border | Cross-sectional | Syrian ,Iraq and Afghanistan refugees  n=6142  All age groups | migrants visiting the field hospital in Dobova | | Cases with coronary artery disease, hypertension, asthma, diabetes | Majority of the patients did not suffer from any tropical disease; no new case of TB was detected; all the tests for HIV, Hepatitis B and C were negative. The spectrum of disease was similar to the one of the host country, i.e. depression, diarrhea, respiratory tract infections and chronic diseases, such as asthma and coronary artery disease in the elderly were common |
| Shortall et al. 2017[33] | On the ferries: the unmet healthcare needs of transiting refugees in Greece | 2016 | Greece | Descriptibe, and prevalence of the users of the PHC on the ferries in Greece. | Cross-sectional | Syrian , Afghanistan and Iraq and other refugees  N=1405  All age groups | Refugees seeking healthcare in the PHC. | | NCD in general | The most common diagnoses were respiratory tract infections, dehydration, nausea and vomiting, and musculoskeletal pain with 39.4% of the disease burden being classified as non-communicable. Exposure to violence was associated with an increased risk of developing mental health issues. 40% of disease burden observed was related to NCDs |
|  |  |  |  |  |  |  |  |  |  |  |

^*^reviews, reports, book-chapters not included
